# Whole-genome sequencing of Western Canadian Borrelia spp. collected from diverse tick and animal hosts reveals short-lived local genotypes interspersed with longer-lived continental genotypes

**DOI:** 10.1099/mgen.0.001276

**Published:** 2024-08-02

**Authors:** Jennifer N. Russell, Min-Kuang Lee, Miguel I. Uyaguari-Diaz, Ashton N. Sies, Danae M. Suchan, William Hsiao, Erin Fraser, Muhammad G. Morshed, Andrew D. S. Cameron

**Affiliations:** 1 Department of Biology, University of Regina, Regina, Saskatchewan, Canada; 2 Institute for Microbial Systems and Society, Faculty of Science, University of Regina, Regina, Saskatchewan, Canada; 3 British Columbia Centre for Disease Control, Public Health Laboratory, Vancouver, British Columbia, Canada; 4 British Columbia Centre for Disease Control, Victoria, British Columbia, Canada; 5 Department of Pathology and Laboratory Medicine, University of British Columbia, Vancouver, Canada

## Abstract

Changing climates are allowing the geographic expansion of ticks and their animal hosts, increasing the risk of *Borrelia*-caused zoonoses in Canada. However, little is known about the genomic diversity of *Borrelia* from the west of the Canadian Rockies and from the tick vectors *Ixodes pacificus*, *Ixodes auritulus* and *Ixodes angustus*. Here, we report the whole-genome shotgun sequences of 51 *Borrelia* isolates from multiple tick species collected on a range of animal hosts between 1993 and 2016, located primarily in coastal British Columbia. The bacterial isolates represented three different species from the Lyme disease-causing *Borrelia burgdorferi sensu lato* genospecies complex [*Borrelia burgdorferi sensu stricto* (*n*=47), *Borrelia americana* (*n*=3) and *Borrelia bissettiae* (*n*=1)]. The traditional eight-gene multi-locus sequence typing (MLST) strategy was applied to facilitate comparisons across studies. This identified 13 known *Borrelia* sequence types (STs), established 6 new STs, and assigned 5 novel types to the nearest sequence types. *B. burgdorferi* s. s. isolates were further differentiated into ten *ospC* types, plus one novel *ospC* with less than 92 % nucleotide identity to all previously defined *ospC* types. The MLST types resampled over extended time periods belonged to previously described STs that are distributed across North America. The most geographically widespread ST, ST.12, was isolated from all three tick species. Conversely, new *B. burgdorferi* s. s. STs from Vancouver Island and the Vancouver region were only detected for short periods, revealing a surprising transience in space, time and host tick species, possibly due to displacement by longer-lived genotypes that expanded across North America.

This article contains data hosted by Microreact.

## Data Availability

DNA sequencing data and genome assemblies are available at the National Center for Biotechnology Information (NCBI), BioProject PRJNA782622 (https://www.ncbi.nlm.nih.gov/bioproject/PRJNA782622).

**Impact Statement**

We present the first genome sequences for *Borrelia burgdorferi*, *Borrelia americana* and *Borrelia bissettiae* from tick hosts *Ixodes pacificus*, *Ixodes angustus* and *Ixodes auritulus* in Western Canada. Whole-genome sequencing of tick-borne *Borrelia* collected from multiple vertebrate hosts and environments over 25 years provides new insights into pathogen diversity and distribution, revealing surprisingly transient populations and the potential replacement of local genetic novelty by widely dispersed types.

## Introduction

Lyme disease is a tick-borne zoonosis caused by spiral-shaped bacterial species belonging to the *Borrelia burgdorferi* sensu lato genospecies complex [1]. As of 2020, 21 species have been named within this complex [2], although only 6 species have been directly implicated as causative agents of Lyme disease (*Borrelia afzelii*, *Borrelia bavariensis*, *B. burgdorferi* sensu stricto, *Borrelia garinii*, *Borrelia mayonii* and *Borrelia spielmanii*) [3]. All members of this complex use ticks in the genus *Ixodes* as vectors, with specific tick and mammal hosts depending on geographical location [4,5]. In Eastern Canada, *Ixodes scapularis* is the primary vector that transmits *Borrelia* to humans [6]; in Western Canada, *Ixodes pacificus* is the primary vector that transmits *Borrelia* to humans [5]. Both tick species are generalists that feed on mammals, lizards and birds [4]. *Ixodes angustus* is geographically widespread across North America; it demonstrates a preference for small mammals and is considered a nest-specific tick; thus, it is rarely implicated in the transmission of *Borrelia* to humans [7]. Tick species *Ixodes auritulus* feeds almost exclusively on birds [8], allowing bird migrations to distribute the tick countrywide [9].

Genetic diversity within *Borrelia* species is strongly associated with the geographic ranges of their animal hosts [5,10, 11]. Profiling these genetic differences is typically performed by multi-locus sequence typing (MLST) of eight chromosomally encoded housekeeping genes (*clpA*, *clpX*, *nifS*, *pepX*, *pyrG*, *recG*, *rplB* and *uvrA*), as the phylogenetic diversity within these genes can be used to infer the evolutionary history of their genomes [10,12, 13]. The outer surface protein gene, *ospC*, encoded on plasmid cp26 is also used to type strains of *B. burgdorferi* s. s. [14,16]. There are currently 21 major *ospC* groups [14], although only 12 have been associated with human infection (types A, B, C, D, E, F, G, H I, K, M and N) [17]. Surveys of natural infections and laboratory-based experiments provide varying degrees of evidence that different *B. burgdorferi* strains are adapted to different host species [18], as postulated in the multiple niche polymorphism hypothesis [19]. Importantly, strains differ in fitness and transmissibility in tick and vertebrate hosts [18], underscoring the importance of surveying natural tick and vertebrate host associations across geographic scales and extended time periods to identify risk factors.

Lyme disease is classified as an emerging disease in Canada due to accelerating host expansion with climate change and increased importation of *Borrelia*-infected ticks on migratory birds [6,9, 20, 21]. The genetic diversity of *Borrelia* and the associations of bacterial genotypes with tick and vertebrate hosts are well-studied in Eastern North America, where Lyme disease is prevalent. However, genotypic diversity and host associations are much less understood in Western North America. The purpose of our study was to expand the understanding of genetic diversity, tick associations and vertebrate associations by genotyping and phylogenetically comparing *Borrelia* isolates collected from west of the Canadian Rockies between 1993 and 2016. This study provides the first *B. burgdorferi* s. s. genome sequences from west of the Canadian Rockies, enabling genomic comparison of *B. burgdorferi* s. s. across Canada.

## Methods

### Sample collection

Infected ticks and mice were acquired between 1993 and 2016 and submitted to the BCCDC. Tick species and animal hosts are summarized in Table 1. All primary specimens were collected within BC, Canada, except for S42, which was submitted to the BCCDC by a veterinarian from the Edmonton metropolitan area in Alberta, Canada. Although the bacterial isolates S50 and S90 were cultured from ticks collected in BC, these strains arrived in the province via known travel from NH and WA states, USA, respectively. Spirochetic isolates (*n*=51) were cultured from mice and tick tissues according to Morshed *et al.* [22].

**Table 1.** The animal and environmental sources of tick species from which *Borrelia* isolates were collected

	Tick species				Tissue culture	Total isolates
**Tick source**	***I.angustus***	***I.auritulus***	***I.pacificus***	***I.scapularis***		
Humans(*Homo sapiens*)	1		4			5
Wood mouse (*Apodemus sylvaticus*)	5		7		3	15
Dogs(*Canis lupus familiaris*)	2		13	1		16
Cats(*Felis catus*)			1			1
Birds (*Aves* sp.)		5				5
Squirrels(*Sciuridae* sp.)	2					2
Environmental			1			1
Flagging			6			6
Total	10	5	32	1	3	51

### DNA extraction and sequencing

DNA was extracted from bacterial cultures using a Qiagen DNeasy Extraction Kit (Qiagen, Germany). Sequencing libraries were constructed using a Nextera XT DNA library preparation kit (Illumina, USA) and assessed for quality using an Agilent Bioanalyzer (Agilent, CA, USA). Whole-genome sequencing was performed on an Illumina MiSeq using a MiSeq Reagent Kit v3 (2×300) (Illumina, Inc., CA, USA).

PCR of MLST loci and Sanger sequencing were performed on three samples for which whole-genome sequencing generated insufficient coverage of one or more MLST targets: for S38, *nifS* and *recG* were sequenced; for S50, *nifS* was sequenced; and for S90, *clpA* was sequenced using primers detailed in Wang *et al.* [23]. The reaction setup consisted of 15 µl of Q5 Hot Start High-Fidelity 2X Master Mix (New England Biolabs, MA, USA), 3 µl of each outer forward and reverse primer (5 µM) and 9 µl of DNA template. Thermocycling conditions were adjusted according to the manufacturer’s recommendations, and annealing temperatures were calculated for each primer set using the Q5 Hot Start High-Fidelity 2X Master Mix product group with the NEB Tm Calculator (v1.15.0). Primer annealing touchdown temperature ranges were 64 –56 °C for *clpA* and 63 °C–55 °C for *nifS*; a stable annealing temperature of 62 °C was used for *recG*. Gel electrophoresis was used to verify the product length and lack of non-specific amplification.

PCR products were purified using the MinElute PCR purification kit (Qiagen, Germany) with a final elution volume of 15 µl in nuclease-free water. DNA concentrations were measured with the Qubit dsDNA Broad Range assay (Thermo Fisher Scientific, USA). Purified PCR products were sent to Eurofins Genomics LLC (Toronto, ON, Canada) for forward and reverse Sanger sequencing using the inner amplicon primers. We were able to improve MLST profiling of these samples by overlapping the Sanger sequences over the gaps in the alignments, as established by the Centre for Genomic Epidemiology MLST profiling software v2.0.9 [24].

### DNA sequence quality control

Between 0.1 and 0.4 million reads were acquired for each *Borrelia* isolate. Sequencing output was quality inspected using FastQC v0.11.8 [25] and MultiQC v1.7 [26]. Adaptor sequences were removed using cutadapt v3.4 [27], and sequences were trimmed and quality filtered (Q20) using Trimmomatic v0.38 [28] with a minimum length of 60 bp. Quality filtering and trimming removed 5.69–27.87 % of reads (Table S1). Trimmed samples had a guanine-cytosine (GC) content of 34–41 % and a read duplication rate of 11.0–33.3 %.

### Genome assembly and isolate classification

Genome assembly was performed using Unicycler v0.4.8 [29], and assembly assessment was performed in Bandage v0.8.1 [30]. Genome assemblies generated 50–437 contigs per genome (Table S2). Taxonomic classification was performed using pyani v0.2.7 [31] with a custom database composed of nine different *Borrelia* reference species [*B. burgdorferi* B31 (ASM868v2), *B. hermsii* CC1 (ASM95631v1), *B. bissettiae* DN127 (ASM22230v1), *B. mayonii* MN14-1539 (ASM193629v1), *B. afzelii* PKo (ASM16559v2), *B. valaisiana* VS116 (ASM17095v2), *B. finlandensis* SV1 (ASM18187v2), *B. spielmanii* A14S (ASM18189v2) and *B. americana* BAA-1877 (ATCC, VA, USA)]. MLST designation was performed through the Centre for Genomic Epidemiology v2.0.9 [24] using the PubMLST database v2023-03-19 [32], and *ospC* typing was performed in blast+ v2.9.0 [33] using the BLASTn function and a custom database composed of 21 *ospC* group alleles; accession numbers for reference sequences can be found in Table S1. MLST types that were perfect matches to the reference database are designated as ‘ST’; MLST types that were imperfect matches to the reference database are designated as ‘NST’. *ospC* types showing complete ORF coverage, but with <92 % nucleotide identity to any reference types, were designated as a novel type. Furthermore, to predict the ORFs for the *ospC* gene in each assembly, NCBI’s ORF Finder was used (RRID:SCR_016643). To identify the location of the cp26 plasmid within each assembly, BLASTn was used, using the cp26 plasmid from *B. burgdorferi* B31 as a reference point; visualization of these plasmids was done using the blast Ring Image Generator (BRIG) v0.95 [34]. For pangenome characterization, genome assemblies were annotated using Prokka v1.14.6 [35], and the pangenome was characterized using Panaroo v1.2.10 [32] using the following parameters: clean-mode strict, remove-invalid-genes and default clustering parameters. All genomes were submitted to PubMLST to be added to their collection of *Borrelia* genomes and to have undefined STs characterized; sample S15 was not defined as an ST due to an incomplete *nifS* locus. Samples were designated by their NST when DNA sequencing did not meet the PubMLST minimum coverage limit for defining new STs. The complete list of PubMLST for each locus is provided in Table S6.

### Phylogenetic analysis

**Fig. 1.**
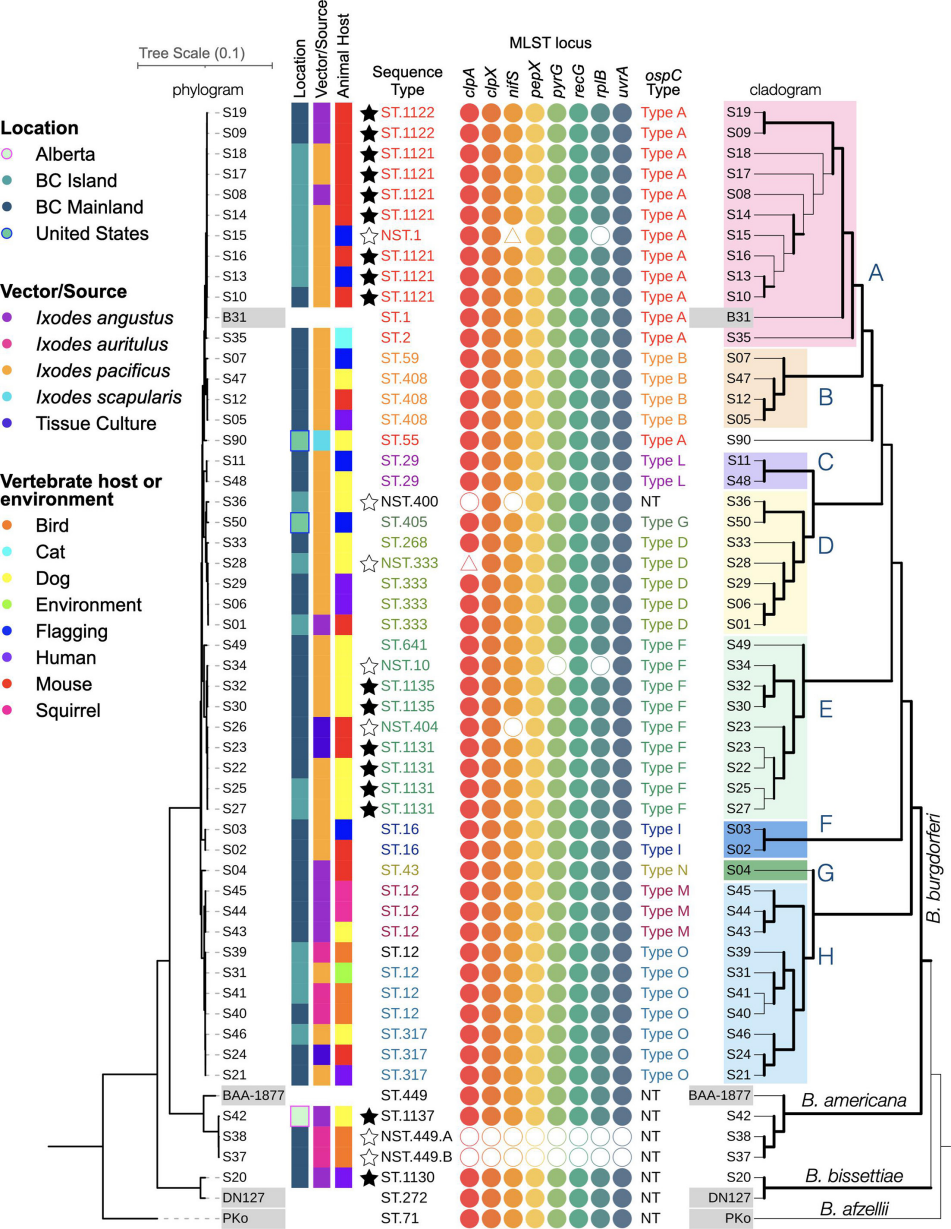
Phylogenetic relationships among *Borrelia* genomes from Western Canada. The phylogeny was generated using BV-BRC’s Bacterial Genome Tree core genes. The same phylogeny is presented as a cladogram on the right, where thick branches indicate bootstrap confidence ≥70 %. Established and newly defined STs are named. Nearest sequence types (NSTs) could not be assigned to STs due to insufficient sequencing coverage of at least one locus. Black stars indicate novel STs where ST designations were generated by PubMLST. Empty stars indicate cases where a ST could not be defined. For each of the eight MLST loci, filled circles indicate a perfect match to an allele in the database, whereas an empty circle indicates a less than 100 % match to a reference allele. An empty triangle indicates incomplete coverage of the locus in the combined whole-genome and Sanger sequencing results. For the *ospC* type, ‘NT’ indicates a novel *ospC* type with <92 % identity compared to defined *ospC* types [52]. Eight subclades in *B. burgdorferi* with high bootstrap support were selected and highlighted with coloured fill in the cladogram to facilitate comparisons in the main text and in later figures. The four reference genomes included in the phylogenetic analysis are highlighted with grey-filled boxes behind their strain numbers.

Phylogenetic analysis of the genomes sequenced in this study (Fig. 1) was conducted using the BV-BRC Bacterial Genome Tree tool [36] using default parameters and the maximal allowable number of genes, in this case 371 core genes. For combined phylogenetic analyses of this study’s genomes with genomes in Tyler *et al.* [13], the nucleotide sequences of *B. burgdorferi*’s eight MLST loci were concatenated in mega11 v11.0.13 [37]. The concatenated sequences were aligned using ClustalW with default settings. Next, maximum likelihood phylogenies were constructed and tested using 1000 bootstrap iterations. For all phylogenetic analyses, the genomes *B. afzelii* PKo (ASM16559v2), *B. bissettiae* DN127 (ASM22230v1), *B. americana* BAA-1877 (ATCC, VA, USA) and *B. burgdorferi* B31 (ASM868v2) were added as references. Phylogenetic trees were visualized using the Interactive Tree Of Life v6.5 [38].

### Comparing genotypes across Canada

To compare and contrast the genetic diversity between *Borrelia* in Western and Eastern Canada, we additionally obtained assemblies from BioProject PRJNA416494 [14], which contains whole-genome shotgun sequences of 64 *B. burgdorferi* s. s. isolates collected in 2016 from Central and Eastern Canada (Manitoba, Ontario and Nova Scotia).

### Statistical analyses

To establish significant links between ST designation and *ospC* designation to genotype, host type and region, Fisher’s exact test statistical analysis was performed in R v4.2.2 [39]. A simulated *P*-value with 2000 replicates was included in this analysis.

## Results and discussion

### Genome assemblies reveal a diversity of novel *Borrelia* sequence types (STs) in Western Canada

Whole-genome sequences were generated by Illumina sequencing of DNA extracts from *Borrelia* isolates cultured at the British Columbia Centre for Disease Control (BCCDC). DNA sequencing and assembly metrics are provided in Table S1, available in the online Supplementary Material. Assembled genomes ranged in size from 888 524 bps (S38) to 1 306 083 bps (S33), with an average genome size of 1 168 949 bps, which is within the current range of *Borrelia* genome assemblies available at the National Center for Biotechnology Information (NCBI) (~0.97–1.52 Mbps). Detailed summaries of each assembly can be found in Table S1.

Average nucleotide identity (ANI) provides robust clustering of closely related genomes, with 95 % or greater nucleotide identity defining members of the same species [40]. ANI clustered 47 genomes with the *B. burgdorferi* s. s. reference genome, 1 genome clustered with the *B. bissettiae* reference genome and 3 genomes clustered with the *B. americana* reference genome (Fig. S1). A whole-genome phylogeny was constructed from alignment of 371 core genes, and tree topology was estimated by maximum likelihood (Fig. 1). A cladogram version of the same phylogeny on the right side of Fig. 1 illustrates that each of the three *Borrelia* species constitutes a high-confidence clade. Furthermore, the ANI and maximum likelihood phylogenetic analysis produced congruent species-level classification of the 51 genomes.

Whole-genome sequences were next classified according to the established sequence typing of *Borrelia* using the PubMLST reference database (v2023-03-19) [41]. Twenty-seven of the isolates were classified into 13 established STs. The remaining 24 isolates were not perfect matches to defined STs (Fig. 1). PubMLST defined six new STs (ST.1122, ST.1121, ST.1135, ST.1131, ST.1137 and ST.1130), which are indicated by black stars in Fig. 1. Seven genomes remained unclassified due to small sequence uncertainties in regions of low coverage; these were named according to a nearest sequence type (NST) and are indicated by empty stars in Fig. 1. Our submissions were the first to the PubMLST database from Western Canada, which partly explains the high number of novel STs identified.

Three isolates contained unusual sequence features at MLST loci. Isolate S03 (ST.16) possessed a single nucleotide deletion at position 228 of the *nifS* locus (Fig. S2), which was confirmed by Sanger sequencing and shotgun sequencing (Fig. S3). The other exceptions were uncertainty in the first 11 nucleotides of the *nifS* locus in S15 (98.05 % overall locus coverage) and the last 18 nucleotides of the *clpA* locus in S28 (96.89 % overall locus coverage); these uncertainties are represented by empty triangles in Fig. 1. Unfortunately, Sanger sequencing of *nifS* and *clpA* did not resolve these uncertainties. In both strains, all sequenced regions were covered at high depth and quality; thus, the NST designations were due only to the two regions of sequence uncertainty.

Many isolates belonged to subclades that correlated with *ospC* type and were supported by high bootstrap values in the whole-genome phylogeny (Fig. 1). We titled these subclades ‘A’ through ‘H’ and highlighted them in the cladogram to facilitate descriptions of Western Canadian *B. burgdorferi* diversity throughout the manuscript. An exception to the correlations between ST and outer surface protein C (OspC) type appeared in subclade H, where ST.317 is nested within ST.12 even though all ST.12 sequences encode identical MLST sequences. The whole-genome phylogeny confidently recognizes ST.317 as a descendent of ST.12, consistent with a single nucleotide substitution in *clpA* at a position in ST.317 where ST.12 has the ancestral allele that matches most other *B. burgdorferi*.

**Fig. 2.**
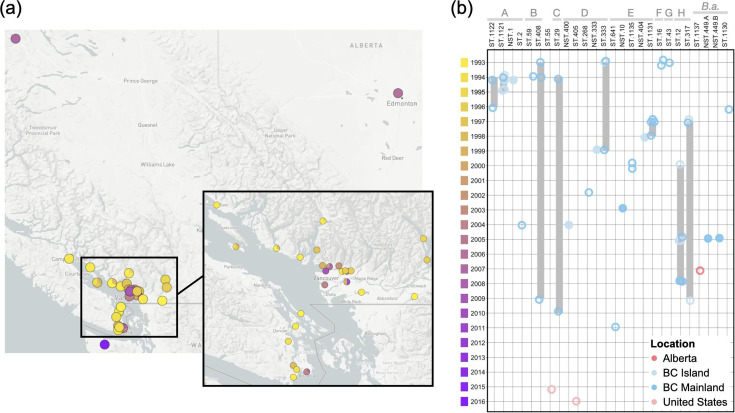
Spatial and temporal distribution of STs across BC. (**a**) Geographic distribution of sample sources plotted with Microreact [46]. Data are available at https://microreact.org/project/gsjARgGQthaR4btaD29wfN-borrelia2024. (**b**) Temporal distribution of STs and NSTs. Blue bars in the plot indicate the timespan in which infected ticks were collected. STs are ordered according to the phylogeny in Fig. 1, and subclades are indicated in grey letters above the STs. STs are indicated by empty circles; NSTs are indicated by filled circles.

**Fig. 3.**
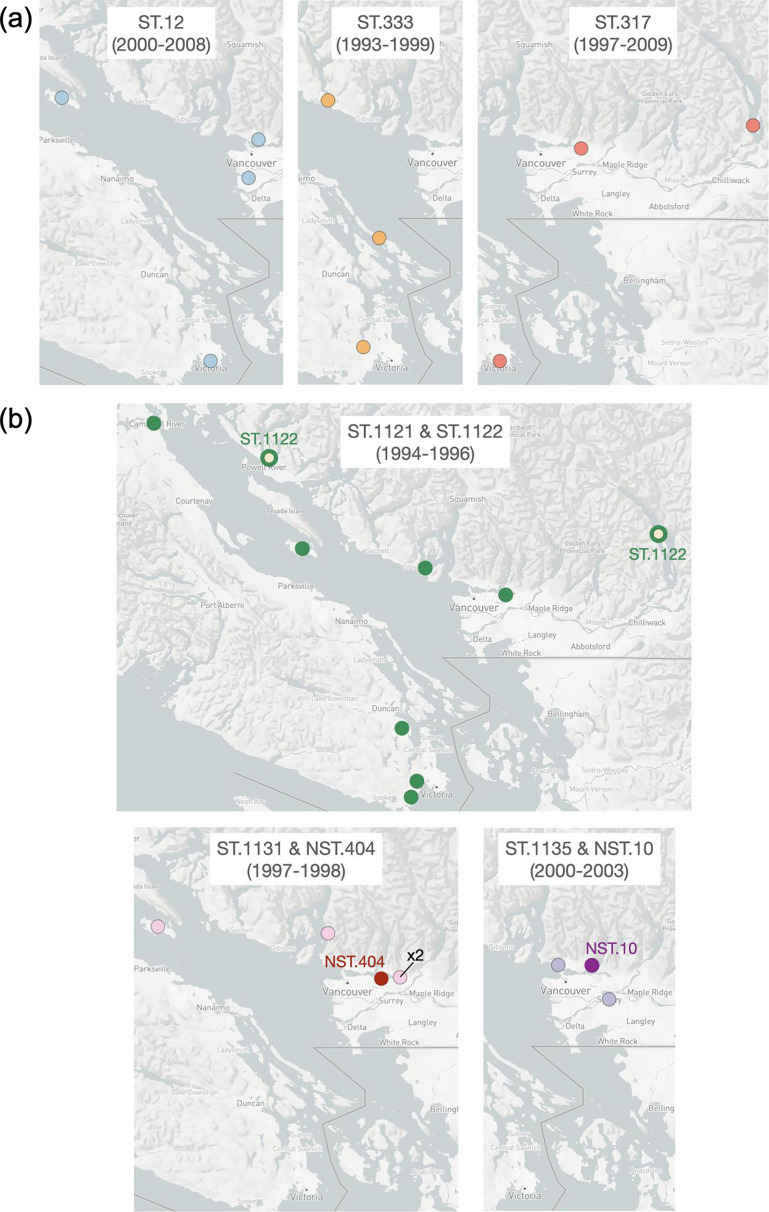
Distribution of select STs and NSTs. (**a**) Distribution of three STs detected over wide geographical ranges and long timespans. (**b**) Distribution of three NSTs over wide geographical ranges within short timespans.

*Borrelia* isolates were cultured from four different tick species that were collected from a diversity of animal hosts and environmental sources, primarily from British Columbia (BC)’s lower mainland and the southern region of Vancouver Island (Figs1  2a). To investigate the relative distribution and persistence of STs over space and time, a temporal plot was generated to accompany the geographical mapping (Fig. 2b). Eighteen STs/NSTs were detected only in a single year, and three additional types were identified in short timespans of 2 to 3 years (Fig. 2b). Three of the long-term STs (ST.12, ST.317 and ST.333) were isolated across the study range on the BC mainland and Vancouver Island (Fig. 3a). In contrast, the new ST.1121 and ST.1122 in subclade A demonstrated broad dispersal but were detected within a short timeframe (Figs2a  3b). Particularly striking was the collection of isolates in subclade A, which contains the *B. burgdorferi* reference strain B31, from ticks on mice or birds. A wide geographic distribution across coastal BC and infection of two tick species, *I. angustus* and * I. pacificus*, did not ensure the persistence of the subclade. Only ST.2 was isolated after 1996 (Fig. 2). Similarly, the STs/NSTs of subclade E were short-lived despite being found in two tick species and two vertebrate species (dogs and mice) (Fig. 3b).

Genome sequencing of one *B. bissettiae* and three *B. americana* isolates is beneficial because few genome sequences are available for these species. *B. americana* was defined based on genetic distances from other *Borrelia* species at multiple loci (16S, *ospA*, p66, *fla* and *rrf-rrl* intergenic sequence) [42]; the ANI distances calculated here are fully consistent with * B. americana* constituting a distinct species within the genus *Borrelia* (Fig. S1). *B. bissettiae* was isolated from an *I. angustus* tick, and *B. americana* was isolated from *I. angustus* and *I. auritulus* ticks; the single previous report of *B. americana* in BC was in *I. auritulus* [42]. Although *B. bissettiae* and *B. americana* were not detected in *I. pacificus*, the small number of * B. bissettiae* and *B. americana* isolates precludes a statistical test of tick preference.

### *ospC* diversity in Western Canada includes a novel *ospC* type

**Fig. 4.**
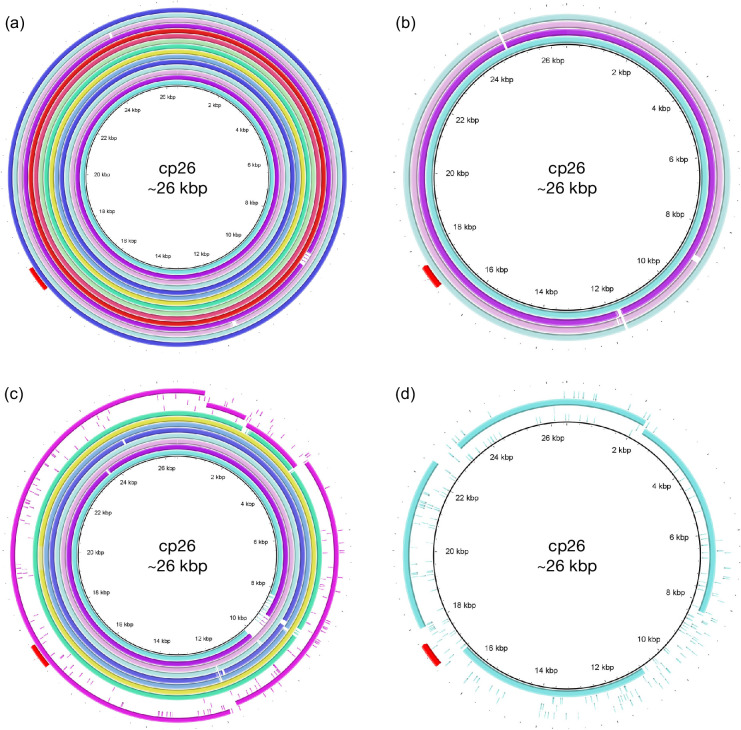
BRIG plots for the *ospC*-containing cp26 plasmid. (**a**) Plasmids encoding type A *ospC*. (**b**) *B. bissettiae* and *B. americana* cp26 assemblies. (**c**) Plasmid assemblies composed of multiple contigs; the outer four rings (pink) are from sample S34. (**d**) Contig mapping to cp26 from sample S39. In all plots, the outermost ring (red) shows the position of the *ospC* ORF. BRIG plots were aligned against the reference cp26 plasmid (AE000792.1) in *B. burgdorferi* B31.

A major determinant of *Borrelia* pathogenicity is the OspC, encoded by the *ospC* gene on the 26 kb circular plasmid cp26 [43]. Among the 51 genomes from BC, complete *ospC* open reading frames (ORFs) were present in 50 assemblies (Figs1 and 4ac), and a partial *ospC* ORF assembled in S39 (Fig. 4d). *ospC* ORFs ranged from 624 to 642 bp, which is within the 620–690 bp range of *ospC* ORFs available on NCBI. For S39, four contigs covered the majority of the cp26 plasmid, although *ospC* was not fully covered by the assembly (Fig. 4d). Mapping S39 sequencing reads against our database allowed us to generate a whole *ospC* sequence, which was type O (Fig. S4). The cp26 plasmid resolved as a single full-length contig in 42 of 51 assemblies (Table S3). Six of 51 assemblies (S01, S04, S12, S38, S46 and S49) assembled the majority of the cp26 plasmid as a single contig, with lengths of 23 390–24 904 bp, each containing *ospC*. In three genomes (S07, S34 and S50), cp26 was split across two or more contigs that together covered the majority of the cp26 plasmid in each strain (Fig. 4c).

Unlike MLST, which categorizes strains into specific sequence variant types, *ospC* typing uses allele groups that are defined by less than 2 % nucleotide difference within a group and greater than 8 % nucleotide difference between groups [19,44]; nucleotide identities above 92 % are classified as the same *ospC* type [15,45]. Forty-six *B. burgdorferi* s. s. isolates could be classified into ten *ospC* types (A, B, D, F, G, I, L, M, N and O). Of the ten *ospC* types identified, eight have been previously linked to Lyme disease incidence (A, B, D, F, G, I, M and N) [17]. Only the *ospC* sequence of isolate S36 did not possess >92 % nucleotide identity to any defined *ospC* type; thus, it is reported as ‘not typed’ (NT) in Fig. 1. The closest match to S36 *ospC* was type A at 86 % identity. The S36 *ospC* ORF was queried against all NCBI submissions belonging to the *Borrelia*/*Borreliella* genus (downloaded on 8 March 2022) using BLASTn. The S36 *ospC* ORF (636 bp) yielded a 100 % match to a 601 bp partial *ospC* coding sequence (JQ308234.1) detected in a chipmunk (*Tamias senex*) native to the Southwestern USA [42,46]; there are currently no full *ospC* ORFs that perfectly match this allele on NCBI.

*B. bissettiae* and *B. americana* also encode *ospC* on cp26, but *ospC* types are not defined in these two species [43,44], which is indicated by ‘NT’ designations in Fig. 1. The *B. bissettiae* sequence aligned most closely to *ospC* type N (88 % identity); the three *B. americana* sequences aligned to *ospC* types A, E and T at~84 % identity (isolates S37, S38 and S42, respectively).

### National and regional MLST types in Canada

**Fig. 5.**
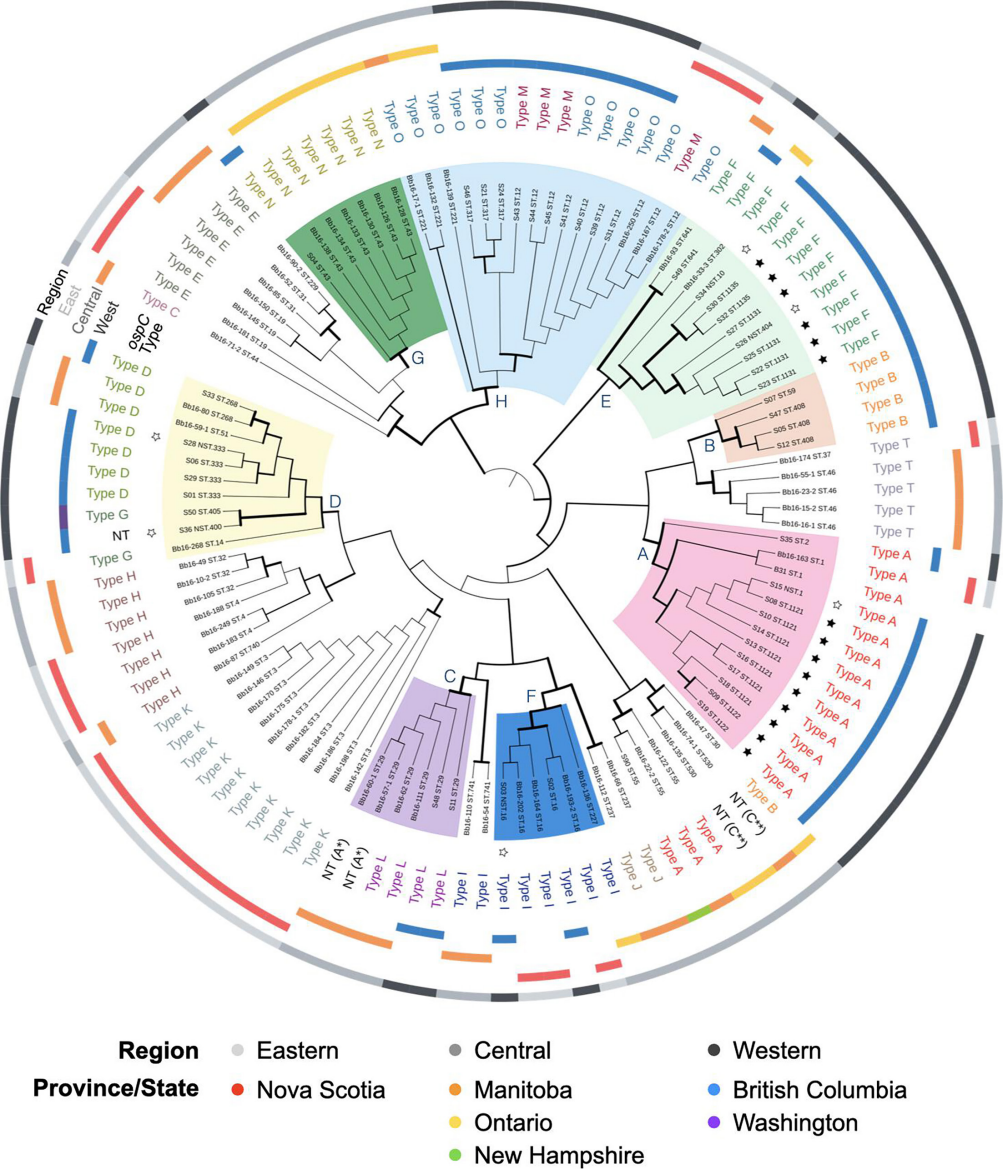
The phylogenetic relationship of *Borrelia* genomes from isolates collected in Western, Central and Eastern Canada. The nucleotide sequences of the eight MLST loci were concatenated for each genome and aligned, and a maximum likelihood phylogenetic tree was constructed. The phylogeny is mid-point rooted on the longest branch, which clusters clades A–F separate from clades G–H, which is the same deepest branch of *B. burgdorferi* observed in Fig. 1. MLST types are indicated within the leaf names; ST denotes a perfect match to an ST, while NST denotes an imperfect match to an ST. Black stars indicate novel STs, where an empty star indicates that the assembly had inadequate coverage of at least one MLST locus for confident ST assignment. ‘NT’ indicates <92 % nucleotide identity to established *ospC* types [45]. Isolates from Central and Eastern Canada are named as in Tyler *et al*. [13]. Four isolates from Central and Eastern Canada are *ospC* type ‘NT’ because they did not meet the >92 % threshold applied in the current study; the *ospC* allele designation (A*, C**) used in Tyler *et al*. [13] is indicated in brackets.

For a pan-Canadian perspective of *B. burgdorferi* diversity and distribution, we applied the same typing schemes to the 64 *B. burgdorferi* genomes from Central and Eastern Canada presented by Tyler *et al*. [13]. This yielded 27 STs and 16 *ospC* types (Fig. 5). A phylogenetic reconstruction of the relatedness of all 109 Canadian genomes found that pan-Canadian STs were distributed across the phylogeny (Fig. 5). Six STs (ST.12, ST.16, ST.29, ST.43, ST.268 and ST.641) were present in both the Western Canada and the Central/Eastern Canada whole-genome datasets. The subclades A, C, D, E, F, G and H of Fig. 1 expanded with the addition of strains from Central and Eastern Canada.

Numerous Western Canadian genomes were not closely related to genomes from Central and Eastern Canada. Subclade B remained a distinct clade containing combinations of ST and *ospC* types that lack Central or Eastern representation in the comparator dataset (Fig. 5). None of the new STs (ST.1121, 1122, 1131 and 1135), NSTs (NST.1, 10, 333, 400 and 404) or five established STs (ST.2, ST.59, ST.317, ST.333 and ST.408) from Western Canada were present in the Central and Eastern Canadian genomes (Fig. 5). All five Western Canadian NSTs were most closely related to STs detected in the USA by Sanger sequencing (Table S5), not to STs identified in Central or Eastern Canada.

**Fig. 6.**
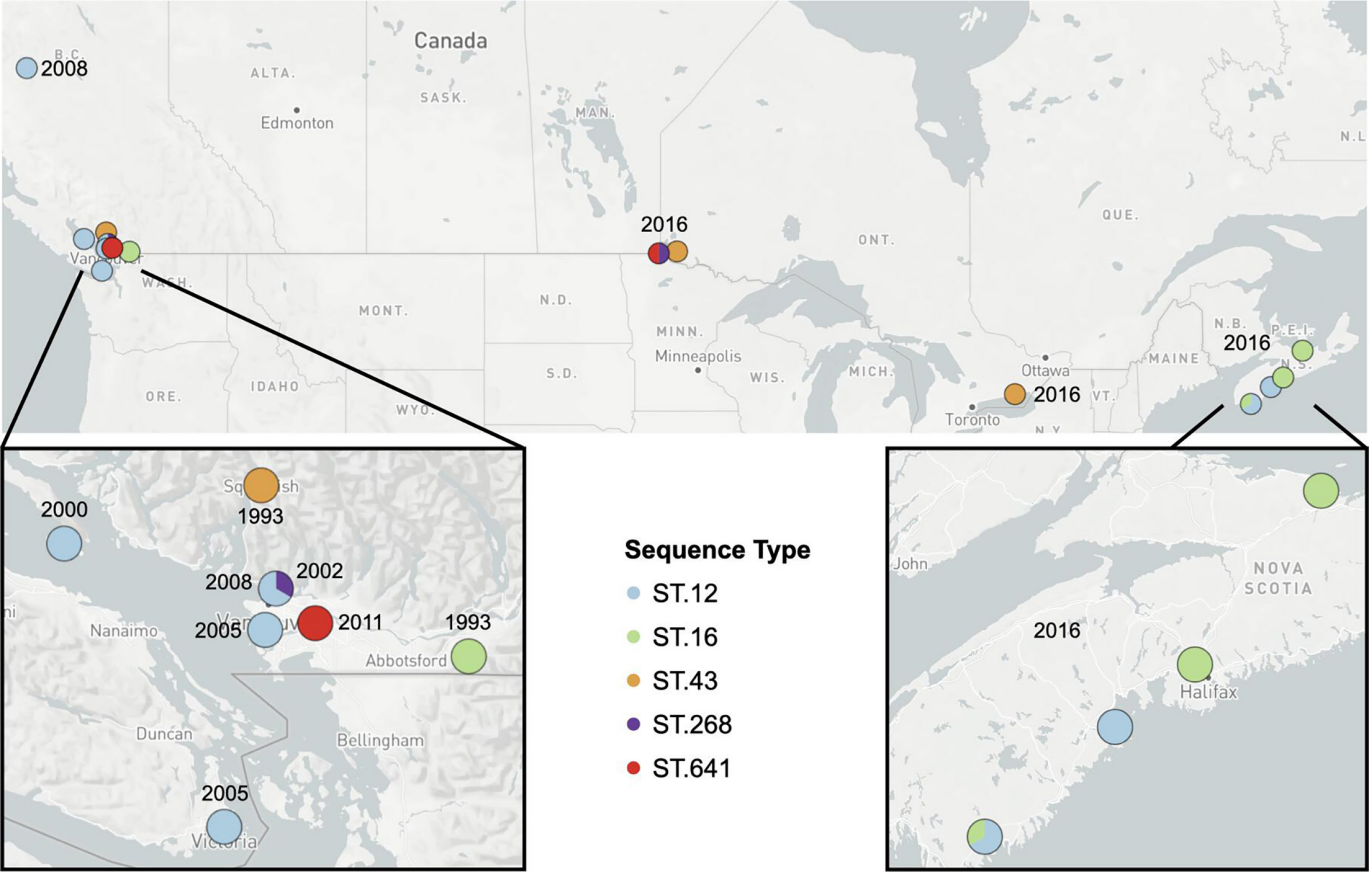
Spatial and temporal distribution of widespread STs identified from whole-genome sequences. Geographic plotting was performed using Microreact^21^ v228.

To expand temporal comparisons, we utilized MLST data from PCR amplicon sequences in the PubMLST database. A caveat of this analysis is that some isolates in the database are date stamped according to the date of sequence submission to the PubMLST database, not the date of primary sample collection or isolation. The most widely distributed ST in BC, ST.12 (2000–2008), was also detected by whole-genome sequencing in Nova Scotia (2016) and by Sanger sequencing in Manitoba (2015–2018), Quebec (2012–2015), Ontario (2012–2019) and Nova Scotia (2002–2016) (Fig. 6 and Table S5). ST.12 has also been detected by Sanger sequencing in the Western, Central and Eastern USA between 1997 and 2016 (Table S5). ST.317, the ST.12 derivative also in subclade H, was not present in the Central and Eastern Canadian genome sequences but has been detected in Quebec and Nova Scotia by MLST.

Four additional STs (ST.16, ST.29, ST.43 and ST.268) have been detected by both whole-genome sequencing and Sanger sequencing across Canada and the USA (Fig. 6 and Table S5). The S90 isolate in our dataset was from NH, USA, clustered with ST.55 isolates from Eastern Canada (Fig. 5).

The Western Canadian *B. burgdorferi* genomes without matching STs in Central and Eastern Canada could be compared to previously defined types in PubMLST. Three STs (ST.2, ST.333 and ST.408) have been detected in CA but not elsewhere in Canada (Table S5). Isolate S50 (ST.405) that we sequenced from WA, USA, is an ST also reported in CA. Numerous STs and NSTs ranged from the Southwestern USA to Western Canada. This range along the Pacific Coast of North America corresponds to the range of the tick host *I. pacificus* and the western flyway of migratory birds in a corridor between the Pacific Ocean and the Rocky Mountains. Northward migration of land birds distributes millions of infected ticks into Central and Eastern Canada each year [47]; an equivalent process in the Pacific flyway likely contributes to *B. burgdorferi* dispersal on the Pacific Coast.

### *ospC* diversity and distribution across Canada

*B. burgdorferi* population genetics is largely clonal [45], but cases of recombination through horizontal transfer of plasmid cp26 can explain *B. burgdorferi* isolates where MLST ST is not predictive of *ospC* type [48,49]. In the Western Canadian genomes, Fisher’s exact test confirmed that MLST ST is predictive of *ospC* type (*P*<0.0005) (Table S4A), consistent with the vertical inheritance of the essential plasmid cp26 that encodes *ospC*. In a small number of cases, *ospC* types occurred outside of a highly supported subclade containing the same *ospC* type(s). In Fig. 1, only S49 (*ospC* type F) and S90 (*ospC* type A) did not group within highly supported clades along with other cases of the same *ospC* types. However, the expanded phylogeny of all 111 *B. burgdorferi* genomes from across Canada grouped both S49 and S90 with other instances of the same *ospC* types.

Almost all *ospC* types detected in Western Canada were also present in whole-genome sequences from Central and Eastern Canada. The sole exception was the novel *ospC* type in isolate S36 (Fig. 5). Conversely, eight *ospC* types (C, E, H, J, K, T, Y and C**) in the Central and Eastern Canada genomes were not detected in Western Canada. This pattern is consistent with the prediction that all Western genotypes are descended from Eastern North America. Fisher’s exact test confirmed a significant correlation between ST (or NST) designation and the province/state where the sample originated (*P*<0.0005) and a significant correlation between *ospC* type and province or state (*P*<0.0005; Table S4B), reflecting how only a subset of * B. burgdorferi* lineages spans the Rocky Mountains.

Two additional relationships were detected by Fisher’s exact test in the Western Canadian *B. burgdorferi* isolates: one between *ospC* and the animal host or environmental source of a tick (*P*<0.001; Table S4A) and another between *ospC* and tick species (*P*<0.0085; Table S4A). The geographically widespread ST.12 was isolated from all four tick species (clade H in Fig. 5). ST.12 and its derivative ST.317 isolated from *I. pacificus* and *I. auritulus* in Western Canada were *ospC* type O, whereas the three isolates of ST.12 from *I. angustus* were *ospC* type M. These distinctions between ST.12 *ospC* types among tick host species in the west potentially represent cases of tick-host specificity. In contrast, Central and Eastern isolates from *I. scapularis* had either *ospC* type M or O (clade H in Fig. 5).

### The pangenome of *B. burgdorferi* sensu stricto in Canada

The pangenome of Western Canadian *B. burgdorferi* s. s. isolates was calculated by Panaroo to contain 1602 genes, of which 581 genes (~36 %) are core genes shared by more than 99 % of isolates (Table 1a). The addition of the Central and Eastern Canadian *B. burgdorferi* s. s. genomes increased the pangenome size to 1740 genes and the number of core genes to 716 (~41 %) (Table 1b). An additional 10 % of the pangenome was classified as ‘softcore’ genes that are present in 95–99 % of all 107 genomes. Thus, the pangenome of Canadian *B. burgdorferi* s. s. is approximately evenly divided between highly conserved genes (core plus softcore) and genes that are sporadically dispersed within the genus.

**Table 2.** Pangenome analysis by Panaroo (v1.2.10; Tonkin-Hill *et al*. 2020 [32]. Core genes are defined as being present in >99 % of genomes, softcore genes are defined as being present in >95 % of genomes, shell genes are defined as being present in >15 % of genomes and cloud genes are defined as being present in >0 % of genomes

(a) *B. burgdorferi* sensu stricto									
	**Totalpangenome (genes)**	**Coregenes**		**Softcoregenes**		**Shellgenes**		**Cloudgenes**	
		**No**	**%**	**No.**	**%**	**No.**	**%**	**No.**	**%**
West (*n*=47)	1602	581	36.0	205	12.8	521	32.5	295	18.4
All (*n*=109)	1740	716	41.1	170	9.8	557	32.0	297	17.0

**(b)All** ***Borrelia*** **spp. sequenced**									
	Total pangenome (genes)	Core genes		Softcore genes		Shell genes		Cloud genes	
		No.	%	No.	%	No.	%	No.	%
West (*n*=51)	1668	466	28.0	255	15.3	584	35.0	363	21.8
All (*n*=113)	1759	644	36.6	207	11.8	590	33.5	318	18.1

## Conclusions

Climate change is increasing the risk of *Borrelia* infection as the ranges of tick and mammal hosts expand in Canada, increasing the importance of finding *Borrelia* reservoirs and understanding whether *Borrelia* STs occupy predictable niches. The common ancestor of North American *B. burgdorferi* was estimated to date to ~60 000 years ago, and its population expanded rapidly after the last glaciation period ~20 000 years ago [50]. The complex phylogenetic structure across North America can be explained by gene flow [50], which is consistent with our detection of MLST and *ospC* types spanning Western, Central and Eastern Canada. The present study benefits from the consideration of archived *Borrelia* isolates dating back to the early 1990s, providing insights into temporal changes in strain types on Canada’s Pacific Coast from a diversity of tick and vertebrate hosts. For example, the closely related ST.12 and ST.317 were detected in BC over an extended period (1997–2009) and are described in eastern *B. burgdorferi* populations. It is highly unlikely that ST.317 arose *de novo* on both sides of the continent; thus, both STs represent gene flow and long-term persistence across Canada.

Several STs and *ospC* types range from the Pacific Coast to the Atlantic Coast of Canada – an immense geographical area greater than the ranges of tick species and most vertebrate hosts. We previously reported *B. burgdorferi* ST.6 and ST.13 in Victoria, BC, in 2021–2022 [51]; both STs were first described in NY state, again demonstrating connectivity between the Atlantic and Pacific coasts. In contrast, novel STs and a novel *ospC* type were detected only in the focal region of our study. None of these novel types were observed to persist over an extended period, despite some being widespread in BC at the time of their isolation. There is no reason to suspect random extinction events of these widespread STs. Instead, local rarity (or extinction) can be more simply explained by displacement of local strains by expanding STs that possess greater transmissibility and/or persistence [18].

## supplementary material

10.1099/mgen.0.001276 Uncited Supplementary Material 1.

10.1099/mgen.0.001276 Uncited Supplementary Material 2.

10.1099/mgen.0.001276 Uncited Supplementary Material 3.

## Abbreviations

BCCDC: British Columbia Centre for Disease Control
GC: guanine-cytosine
MLST: multi-locus sequence typing
NCBI: National Center for Biotechnology Information
