# Supplementary material for: Whole-genome sequencing of Western Canadian Borrelia spp. collected from diverse tick and animal hosts reveals short-lived local genotypes interspersed with longer-lived continental genotypes
Source: Microb Genom. 2024 Aug 2;10(8):001276. doi: 10.1099/mgen.0.001276 (PMC11296321; doi:10.1099/mgen.0.001276)
Supplement: Uncited Supplementary Material 1. [file mgen-10-01276-s001.pdf]

Supplementary Materials

Table S1: Genome sequencing and assembly metrics.

Sample	Number of Reads Passing Filter	Genome Length (bp)	# Contigs	Largest Contig (bp)	Taxonomic Assignment
S01	241,934	1,167,763	190	99,496	*Borrelia burgdorferi*
S02	367,060	1,155,021	215	158,441	*Borrelia burgdorferi*
S03	395,930	1,235,219	326	402,762	*Borrelia burgdorferi*
S04	361,628	1,277,428	324	597,600	*Borrelia burgdorferi*
S05	406,864	1,283,835	343	147,595	*Borrelia burgdorferi*
S06	358,618	1,250,746	269	132,357	*Borrelia burgdorferi*
S07	354,852	1,250,449	437	113,599	*Borrelia burgdorferi*
S08	314,518	1,077,329	88	361,340	*Borrelia burgdorferi*
S09	448,244	1,040,400	78	305,186	*Borrelia burgdorferi*
S10	414,788	1,044,998	60	257,902	*Borrelia burgdorferi*
S11	429,650	1,277,640	251	282,618	*Borrelia burgdorferi*
S12	337,464	1,251,054	306	135,751	*Borrelia burgdorferi*
S13	329,718	1,043,874	64	159,671	*Borrelia burgdorferi*
S14	335,198	1,022,483	50	176,224	*Borrelia burgdorferi*
S15	454,918	1,041,821	83	197,285	*Borrelia burgdorferi*

S16	331,856	1,043,878	63	300,690	*Borrelia burgdorferi*
S17	426,442	1,048,289	70	597,284	*Borrelia burgdorferi*
S18	407,450	1,081,853	135	304,224	*Borrelia burgdorferi*
S19	435,668	1,078,490	69	365,924	*Borrelia burgdorferi*
S20	542,058	1,264,443	364	446,322	*Borrelia bissetti*
S21	574,040	1,235,230	169	268,787	*Borrelia burgdorferi*
S22	396,526	1,211,942	250	100,415	*Borrelia burgdorferi*
S23	427,542	1,252,510	208	283,735	*Borrelia burgdorferi*
S24	378,332	1,252,372	178	65,834	*Borrelia burgdorferi*
S25	333,182	1,218,707	186	111,394	*Borrelia burgdorferi*
S26	369,052	1,183,861	260	96,351	*Borrelia burgdorferi*
S27	593,050	1,234,634	157	217,045	*Borrelia burgdorferi*
S28	308,288	1,184,712	283	101,540	*Borrelia burgdorferi*
S29	612,114	1,277,203	154	771,367	*Borrelia burgdorferi*
S30	374,450	1,260,918	147	142,115	*Borrelia burgdorferi*
S31	442,400	1,239,597	102	370,148	*Borrelia burgdorferi*
S32	497,546	1,223,572	170	535,783	*Borrelia burgdorferi*
S33	724,746	1,306,083	246	557,568	*Borrelia burgdorferi*
S34	459,644	1,112,975	140	61,485	*Borrelia burgdorferi*

S35	555,106	1,187,715	261	99,346	*Borrelia burgdorferi*
S36	318,208	1,179,386	196	120,297	*Borrelia burgdorferi*
S37	339,354	1,137,943	130	118,169	*Borrelia americana*
S38	317,872	888,524	170	98,847	*Borrelia americana*
S39	542,602	1,043,335	270	67,246	*Borrelia burgdorferi*
S40	465,552	1,189,802	131	53,765	*Borrelia burgdorferi*
S41	470,608	1,261,632	140	292,340	*Borrelia burgdorferi*
S42	385,334	1,187,611	90	341,110	*Borrelia americana*
S43	522,146	1,233,625	186	481,682	*Borrelia burgdorferi*
S44	427,338	1,227,238	223	165,333	*Borrelia burgdorferi*
S45	602,656	1,197,273	221	73,838	*Borrelia burgdorferi*
S46	544,794	1,001,528	342	71,009	*Borrelia burgdorferi*
S47	424,094	1,176,317	276	86,643	*Borrelia burgdorferi*
S48	320,910	1,223,824	163	90,855	*Borrelia burgdorferi*
S49	290,204	1,150,553	402	111,263	*Borrelia burgdorferi*
S50	515,888	982,063	371	105,656	*Borrelia burgdorferi*
S90	213,166	1,216,691	125	98,342	*Borrelia burgdorferi*

Table S2: The representative *ospC* alleles used for *ospC* typing.

**OspC Type**	**Accession #**	**Reference**
Type A	AF029860	Ogden *et al.* 2011
Type B	AF029861	Ogden *et al.* 2011
Type C	AF029862	Ogden *et al.* 2011
Type D	AF029863	Ogden *et al.* 2011
Type E	AF029864	Ogden *et al.* 2011
Type F	AF029865	Ogden *et al.* 2011
Type G	AF029867	Ogden *et al.* 2011
Type H	AF029868	Ogden *et al.* 2011
Type I	AF029869	Ogden *et al.* 2011
Type J	AF029870	Ogden *et al.* 2011
Type K	AF029871	Ogden *et al.* 2011
Type L	L42899	Ogden *et al.* 2011
Type M	U01892	Ogden *et al.* 2011
Type N	L42897	Ogden *et al.* 2011
Type O	X84778	Ogden *et al.* 2011
Type P	U91796	Hanincová *et al.* 2008
Type Q	U91790	Hanincová *et al.* 2008
Type R	U91791	Hanincová *et al.* 2008
Type S	U91793	Hanincová *et al.* 2008
Type T	AF065143	Ogden *et al.* 2011
Type U	AF065144	Ogden *et al.* 2011

Table S3: A summary of the cp26 plasmid and the *ospC* ORF from each genome assembly. The average length of cp26 is ~26 kilobases (Byram *et al.* 2004). For genomes that were unable to assemble the cp26 plasmid as one contig, 'Length' is demonstrating the length of the contig which contains the *ospC* gene.

**Sample**	**Taxonomic Assignment**	**cp26 Length (bps)**	***ospC* Length (bps)**
S01	*Borrelia burgdorferi*	24,630	636
S02	*Borrelia burgdorferi*	26,400	633
S03	*Borrelia burgdorferi*	26,506	633
S04	*Borrelia burgdorferi*	24,904	636
S05	*Borrelia burgdorferi*	26,525	636
S06	*Borrelia burgdorferi*	26,411	636
S07	*Borrelia burgdorferi*	13,457	636
S08	*Borrelia burgdorferi*	26,498	633
S09	*Borrelia burgdorferi*	26,498	633
S10	*Borrelia burgdorferi*	26,499	633
S11	*Borrelia burgdorferi*	26,519	633
S12	*Borrelia burgdorferi*	24,658	636
S13	*Borrelia burgdorferi*	26,499	633
S14	*Borrelia burgdorferi*	26,499	633
S15	*Borrelia burgdorferi*	26,499	633
S16	*Borrelia burgdorferi*	26,499	633
S17	*Borrelia burgdorferi*	26,498	633
S18	*Borrelia burgdorferi*	26,498	633
S19	*Borrelia burgdorferi*	26,498	633
S20	*Borrelia bissetti*	26,439	639
S21	*Borrelia burgdorferi*	26,531	630
S22	*Borrelia burgdorferi*	26,505	630

S23	*Borrelia burgdorferi*	26,505	630
S24	*Borrelia burgdorferi*	26,531	630
S25	*Borrelia burgdorferi*	26,505	630
S26	*Borrelia burgdorferi*	26,361	630
S27	*Borrelia burgdorferi*	26,505	630
S28	*Borrelia burgdorferi*	26,465	636
S29	*Borrelia burgdorferi*	26,511	636
S30	*Borrelia burgdorferi*	26,505	630
S31	*Borrelia burgdorferi*	26,420	630
S32	*Borrelia burgdorferi*	26,339	630
S33	*Borrelia burgdorferi*	26,393	636
S34	*Borrelia burgdorferi*	15,355	630
S35	*Borrelia burgdorferi*	26,479	633
S36	*Borrelia burgdorferi*	26,126	636
S37	*Borrelia americana*	26,159	639
S38	*Borrelia americana*	23,390	624
S39	*Borrelia burgdorferi*	NA	NA
S40	*Borrelia burgdorferi*	26,450	630
S41	*Borrelia burgdorferi*	26,530	630
S42	*Borrelia americana*	26,286	642
S43	*Borrelia burgdorferi*	26,537	639
S44	*Borrelia burgdorferi*	26,537	639
S45	*Borrelia burgdorferi*	26,537	639
S46	*Borrelia burgdorferi*	23,745	630
S47	*Borrelia burgdorferi*	26,525	636
S48	*Borrelia burgdorferi*	26,243	633

S49	*Borrelia burgdorferi*	24,875	630
S50	*Borrelia burgdorferi*	19,353	633
S90	*Borrelia burgdorferi*	26,499	633

Table S4: Fisher's Exact Test statistical analysis comparing Sequence Type and *ospC* designation to other metadata variables. A simulated p-value was generated based on 2000 replicates. * Indicates a significant relationship ($P \leq 0.05$).

**A) Western Canada Samples**		
**Statistical Comparison**	**Sequence Type**	***ospC* Type**
**Sequence Type**	--	0.0004998*
***ospC* Type**	0.0004998*	--
**Tick Species**	0.1354	0.008496*
**Animal/Environmental Source**	0.0004998*	0.0009995*
**B) Eastern and Western Canadian Samples**		
**Statistical Comparison**	**Sequence Type**	***ospC* Type**
**Sequence Type**	--	0.0004998*
***ospC* Type**	0.0004998*	--
**Tick Species**	0.0004998*	0.0004998*
**Province**	0.0004998*	0.0004998*

Supplemental figures

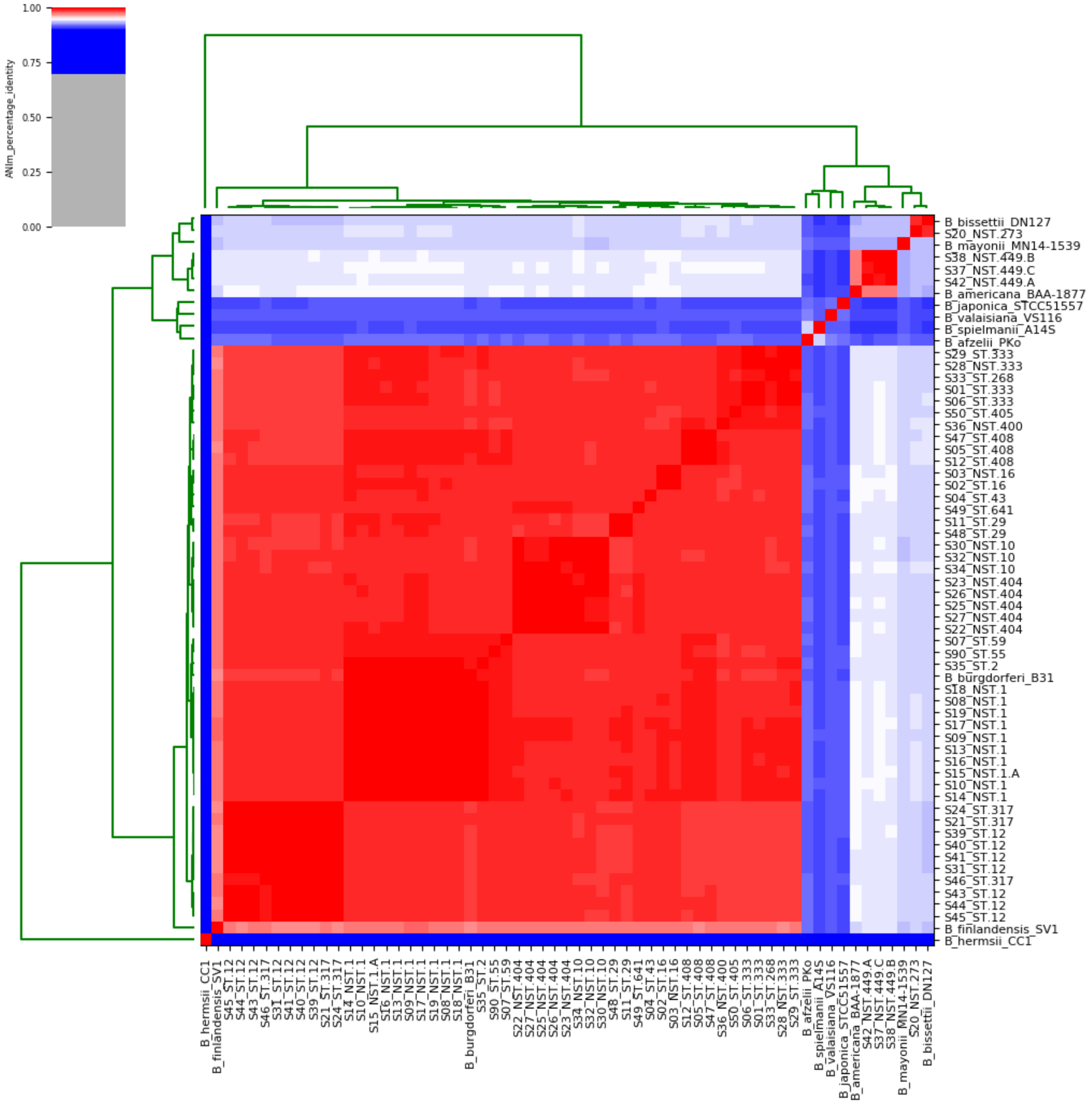

Figure S1: ANI analysis of all 51 genomes sequenced in this study along with reference genomes for seven species spanning the known diversity within the genus *Borrelia*.

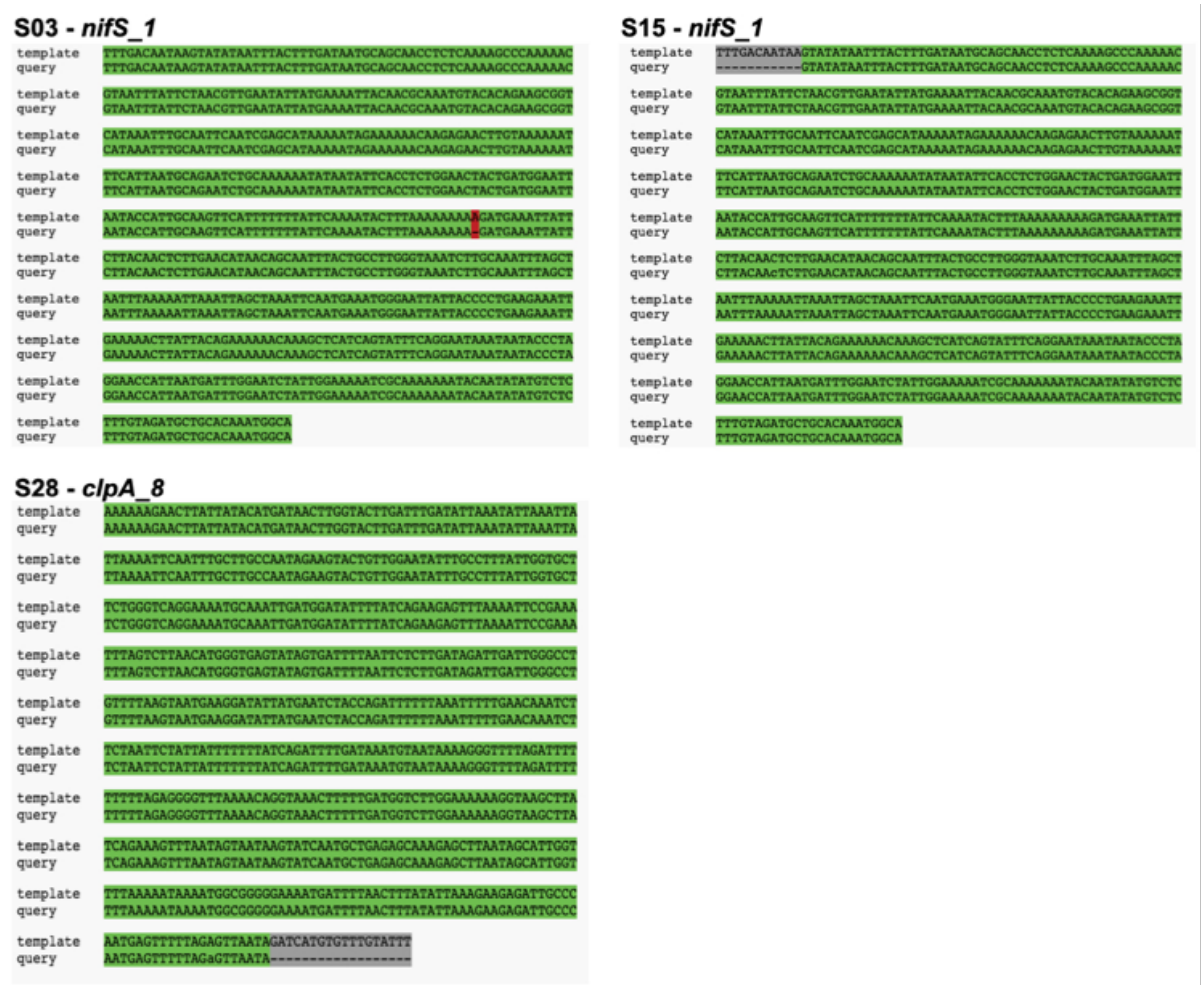

Figure S2: DNA sequences from isolates S03, S15, and S28 where ambiguities arose, as determined in the Centre for Genomic Epidemiology's MLST 2.0 service (v2.0.9; Larsen *et al.* 2012). Isolate sequences are aligned to reference sequences; DNA sequences designated as 'Template' are from the MLST references that most closely matched our sample 'query' sequences.

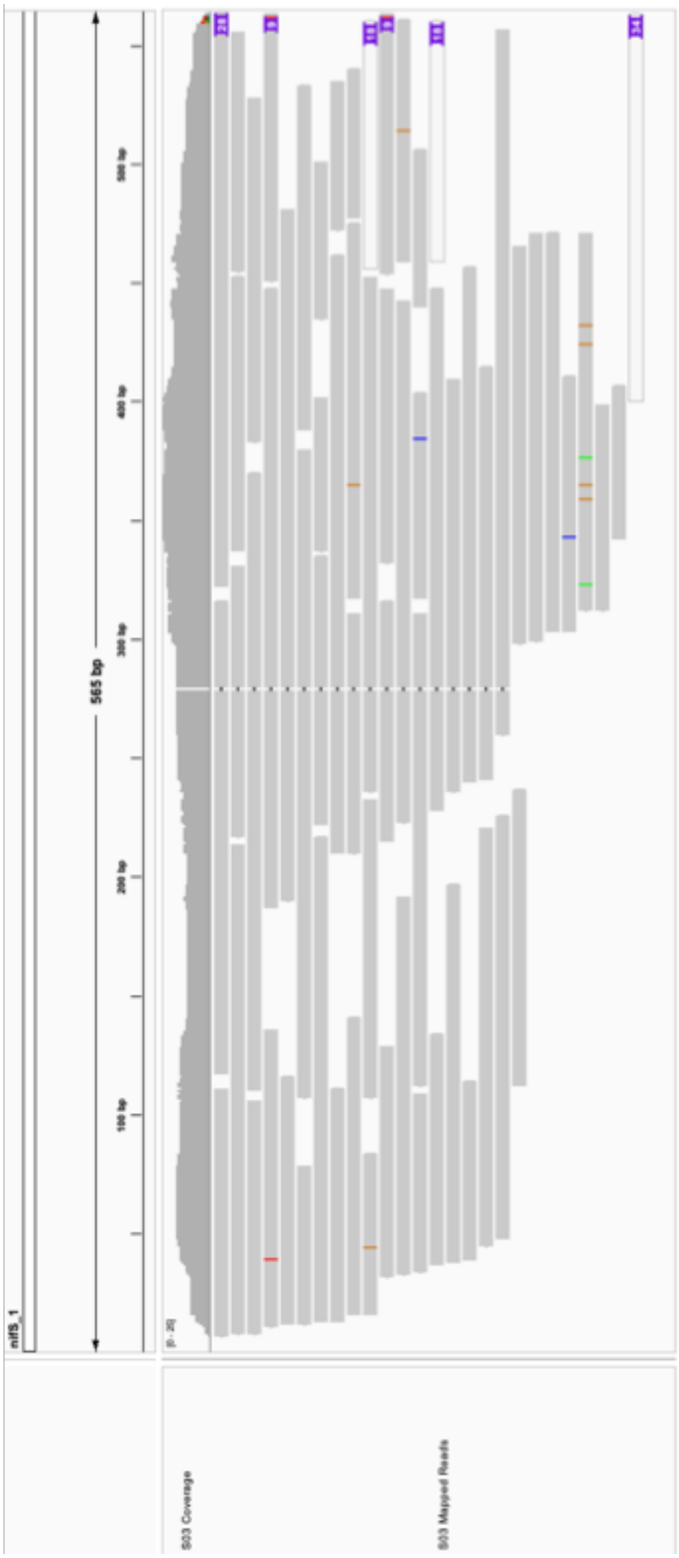

Figure S3: Whole genome shotgun sequencing reads from S03 aligned to the nifS_1 allele for visualization of the single nucleotide deletion in S03.

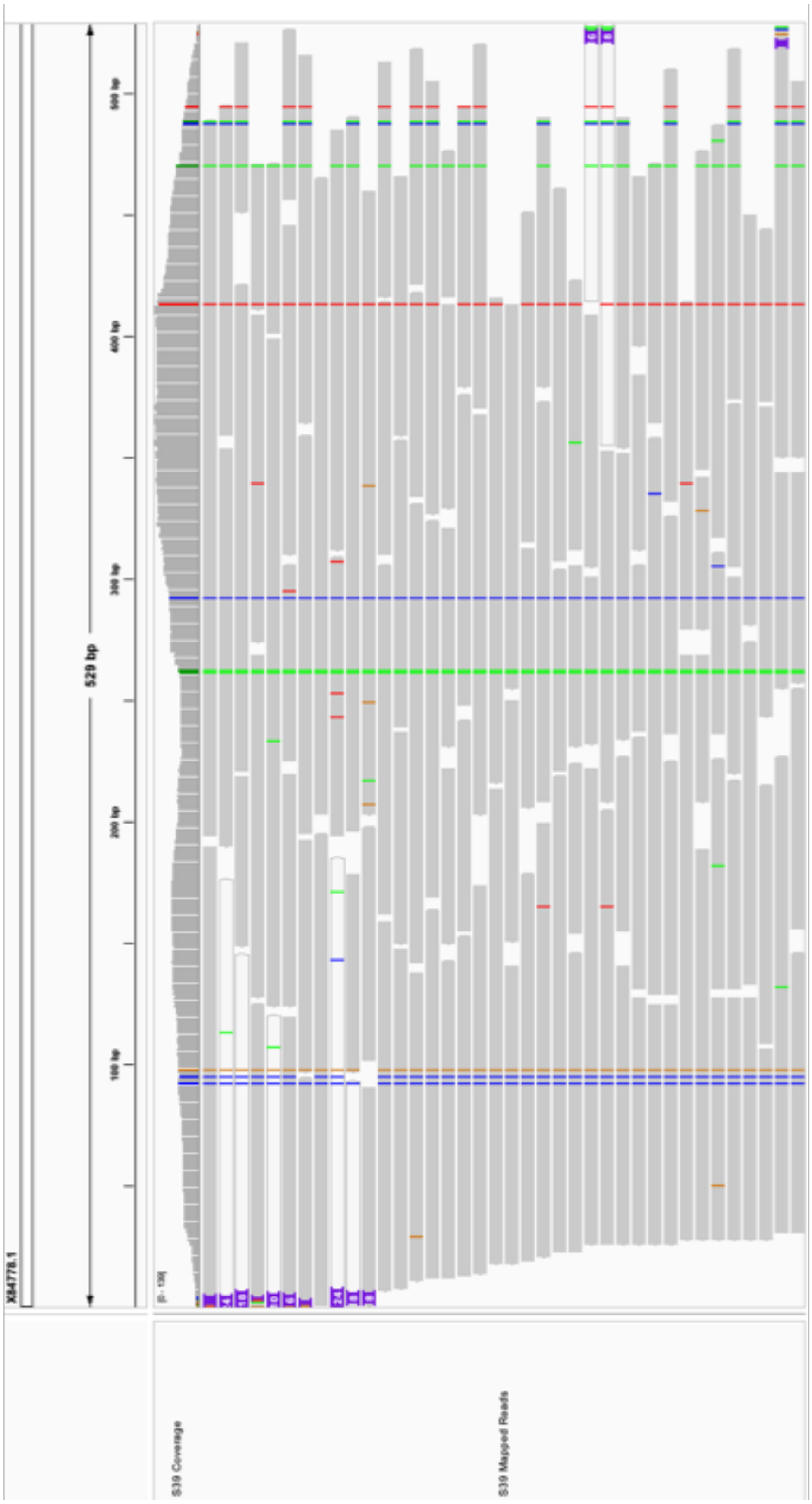

Figure S4: Whole genome shotgun sequencing reads from S39 mapped to the representative *ospC* Type O sequence (X84778.1). Base positions that differ from the reference are coloured.
